# Reproducibility of Proprioceptive Performance in Institutionalized Older Adults Using a Smartphone-Based Joint Position Sense Test

**DOI:** 10.3390/jfmk10040416

**Published:** 2025-10-22

**Authors:** Alejandro Caña-Pino, Alba Marín-Rubio

**Affiliations:** 1 Surgical Medical-Therapy Department, Medicine Faculty and Health Sciences, University of Extremadura, 06006 Badajoz, Spain; amarinru@alumnos.unex.es; 2 Research Group PhysioH (Fisioterapia e Hipoterapia), University of Extremadura, 06006 Badajoz, Spain

**Keywords:** joint position sense, proprioception, smartphone sensors, digital inclinometer, older adults, institutionalized older adults, wearable health technology, postural control, sensorimotor assessment, functional assessment

## Abstract

**Background:** Joint position sense (JPS) is a critical component of proprioception and postural control, especially in older adults, where deficits are associated with increased risk of falls and functional decline. Recent studies have explored smartphone-based digital inclinometers as accessible tools for clinical proprioceptive assessment, but their participant-level reproducibility in institutionalized elderly populations remains unexplored. **Objective:** We aimed to examine the reproducibility of joint position sense performance in institutionalized older adults, using a smartphone-based inclinometer that has been applied in other populations. Assessing joint position sense with accessible smartphone-based tools may provide practical insights for rehabilitation and functional assessment in older adults. **Methods:** Thirty-five-year-old or older adults (mean age 85.9 ± 8.19 years) participated in this test–retest study. JPS was assessed using the iPhone^®^ inclinometer. Participants memorized and attempted to reproduce a 30° forward trunk flexion while standing. The absolute angular error was measured across two sessions, one week apart. Relative and absolute reproducibility were analyzed using intraclass correlation coefficients (ICC 2,1), standard error of measurement (SEM), Smallest Real Difference (SRD), and Bland–Altman analysis. **Results:** The ICC for the whole sample was 0.839 (95% CI: 0.72–0.91), indicating good reproducibility of participants’ proprioceptive performance. SEM and SRD were 3.65° (33.3%) and 10.1° (92.3%), respectively. Bland–Altman plots showed minimal bias (0.23°) and only 5.71% of values fell outside the 95% limits of agreement. **Conclusions:** Participants demonstrated moderate-to-good reproducibility in replicating joint position sense, reflecting consistent proprioceptive performance across sessions. This approach demonstrates feasibility for repeated proprioceptive assessment in this population. These findings have potential applications for functional monitoring and fall-prevention programs in institutionalized older adults.

## 1. Introduction

The aging of the population represents an important socio-health challenge worldwide. In Spain, the proportion of people over 65 years of age already exceeds 20% of the total population, and this proportion is expected to increase significantly in the coming decades [1]. This demographic shift leads to an increase in the prevalence of functional alterations related to musculoskeletal and neuromotor systems, such as loss of strength, decreased balance and impaired proprioception [2,3]. These impairments are directly linked to an increased risk of falls, functional decline, and higher healthcare burden in aging societies.

Proprioception, defined as the ability to detect the position, movement and orientation of joints without visual feedback [4], is essential for postural control and fall prevention. Its deterioration with age affects functionality, autonomy and increases dependency in institutionalized older adults [5]. Therefore, assessing proprioception in this population is not only relevant from a preventive point of view, but also from a therapeutic point of view. Proprioceptive assessment can provide objective metrics for rehabilitation planning and monitoring functional outcomes. Within the proprioceptive components, Joint Position Sense (JPS) has been identified as crucial in the prediction of falls and the design of rehabilitation programs [6,7]. The measurement of Joint Reposition Error (JRE) is commonly used; however, historically, it has required expensive or inaccessible equipment, such as robotic platforms or isokinetic devices [8,9]. The JRE is used as an indicator of how accurately a person can reproduce an initial posture.

Recently, mobile technologies and smartphone sensors have emerged as accessible and cost-effective alternatives [10,11]. Although this traditionally required bulky and expensive equipment, the development of new technologies has allowed access to more practical methodologies. In particular, digital inclinometers integrated into these devices have been applied successfully to assess JPS in the lumbar spine, knee or ankle [12,13,14]. For example, Caña-Pino et al. [12] demonstrated excellent intra-session reliability in measuring lumbar errors using an iPhone^®^ inclinometer, achieving Interclass Correlation Coefficients (ICCs) greater than 0.93 in both standing and sitting positions. Likewise, Chirumbole et al. [15] reported consistent participant performance when assessed with a digital inclinometer app at the hip joint, with ICCs of 0.849 in active testing. In addition, Reddy et al. [16] evaluated the intra- and inter-rater reliability of hip JPS tests with a digital inclinometer, identifying ICCs of 0.88–0.92 in the supine position and 0.64–0.72 in the standing position. Although these findings are promising, there are still gaps to be filled—especially in vulnerable populations such as institutionalized older adults (a group with distinct clinical profiles, higher vulnerability, and unique safety considerations)—regarding the feasibility of conducting proprioceptive assessments and the reproducibility of participant performance under ecological clinical conditions [17]. This is a clinically relevant knowledge gap, as this population would greatly benefit from rapid, objective, and repeatable assessments in their usual environments [5,7,12,17]. Unlike previous studies conducted in younger adults or patients with musculoskeletal conditions, the present work focuses on institutionalized older adults, a vulnerable population where ecological feasibility and reproducibility of proprioceptive assessment have not been explored. Exploring smartphone-based tools in this setting aligns with the current trend toward integrating wearable and mobile health technologies into clinical and rehabilitative practice.

The aim of this study was to evaluate the test–retest reproducibility of proprioceptive performance (joint position sense error) in institutionalized older adults. This was conducted using a smartphone-based inclinometer (iPhone^®^) previously applied in other contexts. Importantly, this study does not validate the device itself but rather assesses the consistency of participant performance under standardized conditions.

## 2. Materials and Methods

The study was registered at ClinicalTrials.gov (registration number: NCT06844578, date: 28 February 2025) and approved by the Bioethics Committee of the University of Extremadura (approval code: 46/2025, date: 21 January 2025). The study was performed following the updates to the Helsinki Declaration, modified by the 64th General Assembly of the World Medical Association (Fortaleza, Brazil, 2013).

### 2.1. Sample Size

The required sample size was estimated a priori following the ICC-based methodology described by Walter et al. [18] and applied by Caña-Pino et al. [12], who reported an ICC of 0.91 (null hypothesis ICC = 0.75) for smartphone-based inclinometer assessment of joint position sense. With α = 0.05, β = 0.20 (80% power), and two repeated measures per participant, the minimum required sample size was calculated as 26 participants.

Nevertheless, we recruited 35 participants to offset potential attrition and missing data, which was within the approved protocol specifying a minimum sample size rather than a fixed maximum. This size is within the recommended range for intra- and inter-session reliability and reproducibility studies using angular measurements with digital sensors.

### 2.2. Participants

Thirty-five institutionalized older adults from a geriatric residential center in Cáceres, Spain, were included. All participants provided a written informed consent prior to inclusion. Inclusion criteria were age 65 years or older, permanent institutionalization in a nursing home (>6 months), ability to stand upright for at least 30 s, and sufficient understanding to follow simple instructions. Participants with severe cognitive impairment (Mini-Mental State Examination < 10), presence of acute or chronic limiting pain [Numerical Pain Rating Scale (NPRS) > 4], severe neurological disorders (e.g., advanced Parkinson’s disease, stroke with severe motor sequelae), or medical contraindication to performing trunk flexion movements were excluded [19]. Of the 35 participants, 21 (60%) had a documented history of at least one fall in the past year, 17 (48.6%) regularly used a walking aid, and 12 (34.3%) had major musculoskeletal diagnoses, including osteoporosis or prior hip fracture. The mean Mini-Mental State Examination (MMSE) score among participants was 21.8 ± 4.6, indicating mild cognitive impairment on average. The most frequent comorbidities were osteoarthritis (57%), hypertension (49%), diabetes mellitus (26%), and osteoporosis (23%). These conditions are representative of institutionalized geriatric populations and may influence proprioceptive and motor performance. Nevertheless, all participants were able to complete the protocol safely, confirming the feasibility of this assessment in real clinical environments.

This study aimed to explore the reproducibility of proprioceptive performance in this meaningful clinical population, using a smartphone-based inclinometer as a practical and accessible tool for functional assessment. Only participants with complete test–retest data were included in the final analysis (n = 35).

### 2.3. Measurements

Several measurements were collected for sample characterization. First, we recorded participants’ age and pain intensity using the Numerical Pain Rating Scale (NPRS). Second, the participant’s bodyweight (kg) and height (cm) were measured without shoes. The main measure of the study was the Joint Reposition Error (JRE), which was operationalized as the absolute angular difference between the reproduced and target position, a widely used marker of proprioceptive performance, irrespective of the specific measuring instrument [9,12].

The Level function integrated into Apple’s native Measure application (iOS 17.2, Apple Inc., Cupertino, CA, USA) was used to record the trunk inclination angle. This feature operates as a digital inclinometer using the device’s internal triaxial accelerometer. The device was placed in the sagittal plane and locked to a single measurement axis (pitch). Before each session, the inclinometer was calibrated by placing the iPhone^®^ on a verified horizontal surface and zeroing the pitch angle using the built-in calibration function. All measurements were displayed in degrees (°) with a precision of 0.1°. This technology has previously been applied in research to assess joint position sense across various body segments, such as the lumbar spine, with high correlation to more complex standard methods [12].

Previous studies have shown that the iPhone^®^ inclinometer (Apple Inc., Cupertino, CA, USA) provides high reliability and reproducibility, with consistent participant performance across different contexts [12,20]. Therefore, this study does not aim to validate the tool itself, but rather to assess the reproducibility of proprioceptive performance using this method in a novel, clinically relevant older adult cohort.

### 2.4. Reproducibility Procedures

**Figure 1:**
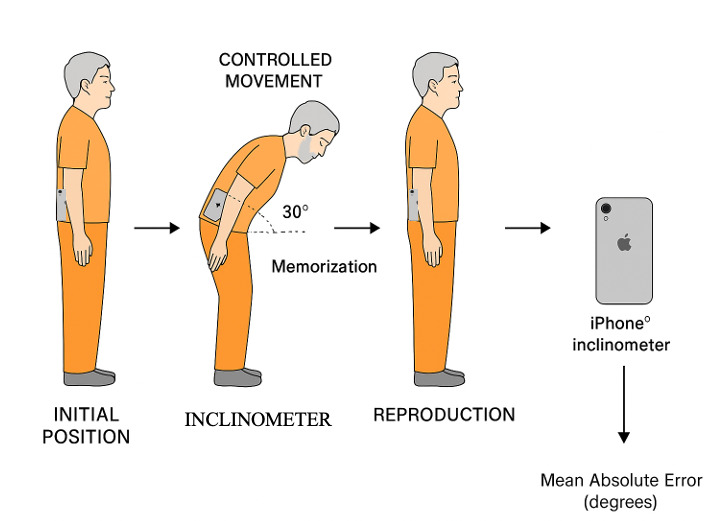
Experimental protocol for joint position sense (JPS) assessment in institutionalized older adults using an iPhone^®^ inclinometer. The device was securely placed at the iliac crest level with the participant standing upright. The smartphone remained attached throughout all test phases (upright, flexion, reproduction). Participants memorized a 30° forward trunk flexion and attempted to reproduce the same position. The inclinometer recorded the angular displacement during both positions.

The test was performed in two sessions separated by an interval of seven days, under controlled conditions, with no changes in the environment, time of day or evaluator. In each session, the procedure was as follows (Figure 1): participants stood barefoot, feet shoulder-width apart, with the iPhone^®^ device secured at the level of the iliac crest using an adjustable belt. The iPhone^®^ was positioned over the right iliac crest using an adjustable elastic belt to align approximately with the L4–L5 segment, which is commonly used as a proxy for global trunk inclination in sagittal plane motion analysis. This placement minimizes upper body obstruction, ensures stable contact with a bony landmark, and allows the inclinometer to capture sagittal trunk movement while maintaining consistency across participants. Although some pelvic contribution is inherent to forward flexion, the task’s limited amplitude (30°) and controlled standing posture minimized pelvic tilt and its influence on the recorded trunk angle. The smartphone’s native inclinometer application was calibrated prior to each session. The participant assumed an upright stance before initiating controlled trunk flexion. Each participant was guided to perform an active forward trunk flexion to an angle of 30°, which was visually displayed on the screen. Once the target position was reached, participants were instructed to memorize it for 10 s before returning to the upright posture. After a standardized 3 s pause, participants attempted to reproduce the same trunk flexion angle without visual feedback. The duration of this pause was kept constant across all participants and both sessions to minimize variability related to memory decay or attentional factors. This protocol was replicated from the study of Caña-Pino et al. [12]. The target angle of 30° forward trunk flexion was chosen based on previous proprioception research and for practical and safety reasons. This range is sufficient to provide a measurable proprioceptive challenge while remaining safe and feasible for institutionalized older adults, minimizing the risk of balance loss or musculoskeletal strain. Preliminary pilot testing confirmed that participants could reliably adopt and maintain this position without discomfort or adverse effects. Two repetitions were performed per session; the first was used as a familiarization trial and excluded from analysis. The first repetition was performed as a familiarization trial and was not included in the analysis. This was performed to minimize potential learning effects and reduce variability caused by initial task novelty, a practice supported in previous proprioception and motor control research, particularly in older adults. The joint repositioning error was calculated as the absolute difference in degrees between the reproduced angle and the target.

The same evaluator, previously trained, applied all the tests to reduce inter-evaluator errors. Participants were asked not to perform vigorous physical exercise or consume caffeine or stimulants in the previous two hours. The data analyst was blinded to the session order during statistical analysis to reduce bias in the interpretation of test–retest results.

The average assessment time per participant was approximately 3–4 min. All participants completed the assessment without any adverse events.

### 2.5. Statistical Analysis

Statistical analysis was performed with the software SPSS.23. The data obtained in the study were subjected to a prior analysis of normality by means of the Shapiro–Wilk test, in order to verify the distribution of the variables. The descriptive characteristics of the sample were expressed as mean and standard deviation. To compare possible statistical differences between test and retest measurements, as well as between sexes, Student’s *t*-test was used for related and independent samples, respectively. A statistical significance level of *p* < 0.05 was established. The assessment of measurement reproducibility included both relative and absolute indicators. Relative reproducibility was quantified using the intraclass correlation coefficient (ICC, model 2,1), based on a two-way random effects model with a single measurement approach and consistency interpretation [21]. A two-way random effects model [ICC(2,1)] with a single measurement and consistency definition was selected, following convention in reproducibility studies where both the participants and measurement sessions are considered random effects.

As the same evaluator and device were used across sessions, an ICC(3,1) model with absolute agreement could also have been applied. However, a sensitivity analysis using this alternative model yielded nearly identical estimates (ΔICC < 0.02), confirming that the choice of model does not materially affect the conclusions.

ICC values were classified using the following established thresholds: below 0.5 (poor), between 0.5 and 0.75 (moderate), 0.75 to 0.9 (good), and above 0.9 (excellent). To evaluate absolute reproducibility, the Standard Error of Measurement (SEM) and Smallest Real Difference (SRD) were calculated using standard formulas derived from the ICC and pooled standard deviation [22]. The SEM was estimated with the formula: SEM = SD × √(1 − ICC), where SD is the mean SD of the two repetitions. The SRD formula was SRD = 1.96 × √2 × SEM. To allow comparison between studies or instruments, SEM% and SRD% were also calculated, expressing both as a proportion with respect to the average of the tests. These values make it possible to determine whether an observed difference can be considered real and compared with other instruments that assess proprioceptive deficits [23]. Both SEM% and SRD% were calculated relative to the grand mean of the two measurement sessions, not the theoretical target angle (30°), in accordance with prior reliability and reproducibility research conventions [23]. Finally, the Bland–Altman analysis was applied to study the level of agreement between the two sessions. In these graphs, the *X*-axis represents the mean of both measurements (test and retest) and the *Y*-axis shows the difference between them. Bias and limits of agreement (LoA) were calculated at 95%. A bias close to zero, together with narrow limits of agreement, is interpreted as a high level of agreement between measurements [24]. To enhance the robustness of the reliability estimates, given the modest sample size, 1000 bootstrap resamples were performed to generate bias-corrected 95% confidence intervals for ICC and SRD values. These bootstrapped CIs closely matched the classical confidence intervals, confirming the stability of the estimates.

Analyses were performed exclusively on participants with complete paired measurements. No imputation of missing values was conducted, as the assessment of test–retest reproducibility requires matched observations from both sessions.

It is important to note that, in this study, test–retest reproducibility reflects the participants’ proprioceptive and motor control abilities, rather than device precision, especially given the clinical frailty and possible neurological variability of the cohort.

## 3. Results

A total of 35 institutionalized older adults were included in the final analysis (men: n = 11; women: n = 24). Table 1 displays the demographic and clinical characteristics of the overall sample, as well as the stratified data for men and women participants.

Joint repositioning error presented a mean of 11.06° (SD = 8.94) in the first session and 10.83° (SD = 9.23) in the second session. No statistically significant differences were observed between the two measurements (t = 0.197; *p* = 0.845). The medians were 9° [Interquartile Range (IR) = 11] and 8° (IR = 9), respectively. The stratified analysis by sex showed the following trends: in men, the mean JRE increased from 9.09° (SD = 9.59) to 11.5° (SD = 12.3) between sessions, without reaching statistical significance (*p* = 0.099), although an upward trend was observed. On the other hand, in women, the JRE slightly decreased from 12.0° (SD = 8.69) to 10.5° (SD = 7.72), without significant differences (*p* = 0.367) (Table 2). These sex-based variations underscore the importance of interpreting proprioceptive performance within subgroup contexts, especially given the larger standard deviations observed in the women’s group.

Table 3 shows relative reproducibility (ICC) and absolute reproducibility (SEM, SEM%, SRD, and SRD%). The intraclass correlation coefficient (ICC 2,1) was 0.839, with a 95% confidence interval of 0.72–0.91, indicating good inter-session reproducibility.

Test–retest reproducibility analysis stratified by sex revealed notable differences. In the men group (n = 11), the intraclass correlation coefficient was exceptionally high (ICC = 0.961; 95% confidence interval (CI): 0.93–0.98), indicating excellent reproducibility. Absolute reproducibility values were equally favorable, with an SEM of 2.16° (21.0%) and an SRD of 5.99° (58.2%), suggesting low individual variability and consistent participant performance.

**Figure 2:**
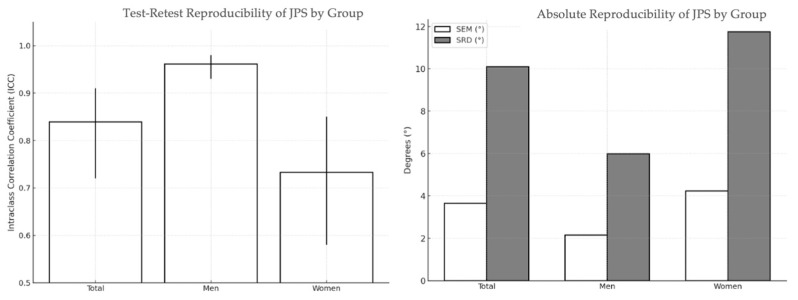
Test–retest reproducibility of joint position sense (JPS) error in institutionalized older adults. Mean intraclass correlation coefficients (ICC 2,1) with 95% confidence intervals are shown for the whole sample, men, and women. Standard error of measurement (SEM) and Smallest Real Difference (SRD) values are also provided to illustrate measurement precision and variability.

In contrast, in the women group (n = 24), reproducibility was moderate to good, with an ICC = 0.733 (95% CI: 0.58–0.85). The SEM was 4.24° (37.7%) and the SRD was 11.75° (104.5%), reflecting a greater dispersion in the individual measurements (Table 3; Figure 2).

A sensitivity analysis excluding extreme values (>2 SD from the group mean) yielded ICCs that remained virtually unchanged (overall ICC = 0.842; men = 0.957; women = 0.726), suggesting that subgroup differences were not driven by outliers or range restriction. Reliability across tertiles of baseline error also showed consistent ICCs (0.81–0.86), confirming the stability of results across different performance levels.

**Figure 3:**
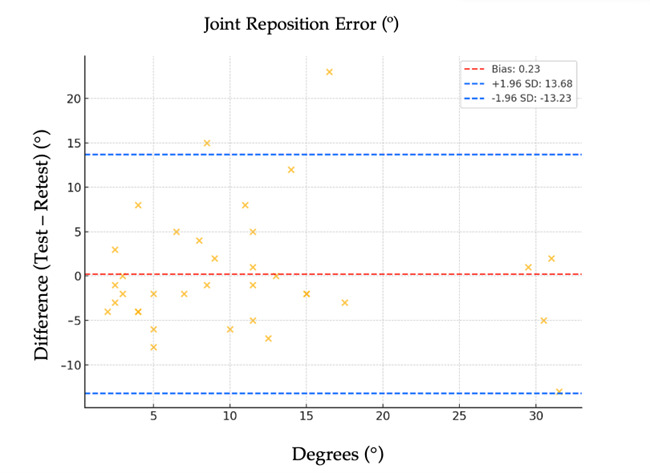
Bland–Altman plot of Joint Reposition Error (JRE) in institutionalized older adults. The *Y*-axis represents the difference between test and retest values (Test–Retest), with positive values indicating higher errors in the second session. The *X*-axis represents the mean error (°) of test and retest trials. Solid lines show the mean bias, and dashed lines indicate the 95% limits of agreement.

To further assess measurement agreement, a Bland–Altman plot was constructed (Figure 3). The analysis revealed a mean bias of 0.23°, with 95% limits of agreement ranging from −13.23° to +13.68°. Approximately 5.7% of data points fell outside these limits, which is consistent with the expected proportion by construction (≈5%). No proportional bias or heteroscedasticity was observed upon visual inspection, indicating homoscedastic measurement error and absence of systematic bias.

## 4. Discussion

This study evaluated, for the first time, the test–retest reproducibility of the joint repositioning error (JRE) using a digital inclinometer integrated into an iPhone^®^ among institutionalized older adults during standing trunk flexion.

Importantly, this study did not evaluate the concurrent validity of the iPhone^®^-based inclinometer against a gold-standard system. Therefore, conclusions about the tool’s accuracy should be interpreted with caution. Our findings primarily highlight the feasibility and reproducibility of applying this method in institutionalized older adults, rather than validating the device per se.

The results showed good reproducibility (ICC = 0.839; 95% CI: 0.72–0.91), an SEM of 3.65° and an SRD of 10.1°, values comparable to those obtained in previous research in populations with chronic low back pain [12]. The mean bias was small (0.23°) and the limits of agreement (−13.23° to +13.68°) were relatively narrow, indicating acceptable consistency between sessions. These findings align with previous research using smartphone technology for proprioceptive assessment in younger or outpatient populations. However, the novelty of the present study lies in its application to an older, clinically fragile, and institutionalized population, under conditions that closely simulate real functional demands, such as standing posture. This context presents additional challenges for sensorimotor evaluation, which reinforces the value of the current results.

Our results agree favorably with previous studies that have validated smartphone sensors in proprioception measurement in young adults with low back pain. For example, Caña-Pino et al. [12] reported an ICC greater than 0.93 in lumbar JRE measurement under similar conditions (standing position) with the same digital inclinometer. Additionally, investigations in hip and knee have recorded ICC between 0.75 and 0.90 and SEM around 3–5°. Al Saadawy et al. [25] demonstrated that a mobile app had excellent ICCs (0.87–0.97 for passive JPS and ~0.62 for active JPS) in a sample with and without osteoarthritis, also showing good concurrent validity with respect to an isokinetic dynamometer. Nakashima et al. [20] performed a direct comparison between the native iPhone^®^ “Measure” app and the VICON system in healthy young people, finding very high ICCs (0.969) and minimal differences at angles of 30° and 60°, showing that smartphone technology can achieve comparable reproducibility in younger populations when tested against reference methods. On the other hand, a study in adults with hip osteoarthritis (n = 62, mean 67.5 years) used a digital inclinometer to assess joint position in standing and recumbency. It obtained very good reliability for flexion and abduction in recumbency (ICC = 0.88–0.92) and good in standing (ICC = 0.64–0.72; SEM ≈ 0.06–0.08) [16]. Chirumbole et al. [15] proposed a digital inclinometer app to measure hip, knee and ankle proprioception. They reported moderate to good reliability for the ankle (ICC = 0.785; 95% CI: 0.539–0.893). In agreement, Lee et al. [26] validated the application in dorsiflexion/plantar flexion, achieving ICCs of 0.79–0.82. These studies support that, even in more technically demanding joints such as the ankle, smartphone-based inclinometers achieve a balance between accessibility and reproducibility; in our case, participant performance remained consistent under ecological conditions. Unlike previous studies in younger or non-institutionalized clinical populations, our findings provide the first evidence on the feasibility and reproducibility of joint position sense assessment in institutionalized older adults, expanding the applicability of smartphone-based tools to this high-risk group. In this sense, the present study confirms that participant proprioceptive performance was reproducible even in a heterogeneous and clinically fragile geriatric population. The widespread use of smartphones among healthcare professionals has expanded the potential for integrating these devices into clinical decision-making processes [27]. The findings of the present study are particularly relevant as they demonstrate that such technology is not only feasible but also effective in older populations with higher levels of frailty and functional variability. Unlike previous research conducted in young or outpatient cohorts, this study confirms the tool’s reproducibility under more challenging real-world conditions. The standing protocol, applied in a population residing in long-term care institutions, enhances the ecological validity of the results and broadens the scope of clinical application for wearable or mobile proprioceptive assessment tools.

When results were analyzed by sex, reproducibility in men was excellent (ICC = 0.961; 95% CI: 0.93–0.98), with low levels of absolute error (SEM 2.16°; SRD 5.99°). In contrast, women presented somewhat lower reproducibility (ICC = 0.733; 95% CI: 0.58–0.85), with higher error rates (SEM 4.24°; SRD 11.75°). This disparity could be explained by factors such as differences in motor control, trunk strength, postural patterns, and pre-existing musculoskeletal conditions that are more prevalent among older women. While the results in women remain clinically useful, the greater individual variability suggests that more standardized measurement protocols or multiple repetitions are required to increase reproducibility. These sex-based differences may also reflect distinct neuromuscular aging trajectories, as older women often present with lower muscle mass, reduced trunk strength, or a higher prevalence of musculoskeletal comorbidities. Additionally, some studies suggest greater intra-individual variability in motor control and balance among older women, which could explain the higher SEM and SRD observed in this subgroup. Future studies should explore whether tailored instructions, adapted postures, or increased familiarization improve reproducibility in women.

Thus, while the method demonstrates good reproducibility for assessing proprioceptive performance, its clinical applicability should focus on detecting substantial, clinically meaningful changes rather than subtle short-term fluctuations. This distinction is important for designing interventions and interpreting outcomes in geriatric populations, where within-subject variability can be high.

Finally, the SRD observed in this study (10.1°, corresponding to approximately 92% of the mean JRE) indicates substantial between-session variability. This magnitude implies that, although the method demonstrates good reproducibility at the group level, it may have limited sensitivity for detecting small or moderate changes in proprioceptive performance at the individual level. Therefore, only changes exceeding the SRD threshold should be interpreted as true changes beyond measurement error, while smaller variations should be considered within the expected variability range. This limitation likely reflects both participant-related neuromotor variability and the ecological testing conditions inherent to institutional care settings.

### 4.1. Strengths and Limitations

This study has several methodological strengths. These include the use of a widely available, portable, and low-cost tool and a digital inclinometer integrated into an iPhone^®^, which enhances its clinical and community applicability. In addition, the protocol was designed under real functional conditions (standing position). Data collection was performed by a single trained evaluator, reducing inter-rater variability, and robust indices of reproducibility were applied, including both relative (ICC) and absolute (SEM, SRD) parameters, with analyses stratified by sex.

However, the study also has limitations. The sample size was moderate and recruited by convenience, which restricts the generalizability of the results. Participants were heterogeneous in terms of functional status but homogeneous in origin (single residence). Most importantly, the study did not evaluate the technical accuracy or intra-device reliability of the iPhone^®^ inclinometer against a gold-standard system (e.g., motion capture or isokinetic platforms). Therefore, our results cannot be interpreted as a validation of the instrument itself. Rather, they strictly describe the reproducibility of participant performance under standardized clinical conditions, using a tool previously validated in other populations. Furthermore, the assessment was limited to a single movement direction (forward trunk flexion), which reduces generalizability. Future studies should incorporate multiple movement planes (flexion, lateral tilt, rotation) and explore additional joints (e.g., knee, ankle, shoulder), along with comparisons to gold-standard technologies to establish concurrent validity. Furthermore, only one analyzed reproduction was performed per session, which may have contributed to the observed measurement variability. Future studies should explore whether averaging multiple repetitions per session reduces SEM and SRD, thereby enhancing measurement precision.

Another limitation is that only one analyzed repetition per session was used after a familiarization trial. Averaging multiple repetitions might reduce intra-individual variability and improve precision; however, this decision was made to minimize fatigue and maintain participant safety in this frail population.

Finally, prospective longitudinal designs are warranted to examine sensitivity to change following interventions such as therapeutic exercise or proprioceptive training. The inclusion of cognitive and functional measures, and subgroup analyses (e.g., by sex, level of dependence, or cognitive impairment), would allow the development of more precise risk and intervention profiles, improving the personalization of geriatric care.

### 4.2. Clinical and Practical Implications

These results carry important implications for clinical practice in geriatric care settings. The use of digital inclinometers integrated into smartphones represents a feasible and reproducible method for measuring joint position sense for assessing proprioceptive performance in institutionalized older adults [28]. Its low cost, ease of use and portability allow its incorporation both in nursing homes and in physiotherapy consultations or fall prevention programs. In addition, knowing the joint repositioning error objectively can help to identify patients with proprioceptive impairment, guide personalized exercise programs and monitor functional evolution over time.

Having a clearly defined threshold for the smallest detectable difference enables clinicians to distinguish normal fluctuations from genuine functional gains following intervention. Also, the use of a functional range (30° in standing) brings the measurement closer to the demands of everyday postural control, which may increase the diagnostic and prognostic utility of the instrument. Furthermore, incorporating this type of assessment into geriatric evaluations may enhance multidisciplinary decision-making, allowing professionals to identify subtle proprioceptive declines before they translate into functional impairment [29]. Given the minimal equipment required, this method could be implemented even in settings with limited access to advanced technology, thereby promoting equity in functional screening across diverse care environments.

## 5. Conclusions

This study demonstrates the reproducibility of proprioceptive performance (JRE) in institutionalized older adults under standardized clinical conditions, using a smartphone-based inclinometer previously validated in other populations. Importantly, this research does not assess or validate the measurement accuracy of the inclinometer itself. The findings support the feasibility of implementing this method in geriatric clinical or educational settings, where consistent replication of joint positioning may reflect meaningful proprioceptive capacity. Only changes exceeding the smallest detectable difference (SRD = 10.1°) can be interpreted as real changes beyond the measurement error.

## Figures and Tables

**Table 1:** Characteristics of the study participants.

Measurements	Total (n = 35) Mean (SD)	Men (n = 11) Mean (SD)	Women (n = 24) Mean (SD)	*p* Value *
Age (years)	85.9 (8.19)	84.8 (10.4)	86.4 (7.15)	0.60
Height (cm)	159 (8.76)	163 (10.3)	157 (7.22)	0.15
Weight (kg)	63.9 (11.9)	61.6 (11.1)	65.0 (12.3)	0.44
Intensity of pain perception (NPRS) (0–10)	1.31 (1.32)	1.18 (1.25)	1.38 (1.38)	0.70

kg: kilograms; NPRS: Numerical Pain Rating Scale. The scale ranges from 0 (“no pain”) to 10 (“worst imaginable pain”); cm: centimeters; SD: standard deviation; * Student’s *t*-test; *p* < 0.05: statistical significance.

**Table 2:** Summary of Joint Repositioning Error (JRE) across test and retest sessions.

Joint Repositioning Error (°)					
	Test (Day 1)		Re-Test (Day 2)		
Test Measurement (°)	Mean (SD)	Median (IR)	Mean (SD)	Median (IR)	*p* Value *
All participants	11.06 (8.94)	9 (11)	10.83 (9.23)	8 (9)	0.845
Men	9.09 (9.59)	6 (9.50)	11.5 (12.3)	6 (8.50)	0.099
Women	12 (8.69)	9.50 (9)	10.5 (7.72)	8.50(8.50)	0.367

°: degrees; SD: Standard Deviation; * Student’s *t*-test.; *p* < 0.05: statistical significance; IR: interquartile range.

**Table 3:** Test–Retest Reproducibility of Joint Repositioning Error (°).

Total (n = 35)	Joint Repositioning Error (°)				
Assessed Action	ICC (95% CI)	SEM (°)	SEM (%)	SRD (°)	SRD (%)
Joint Repositioning Error (°)	0.839 (0.72–0.91)	3.65	33.3	10.1	92.3
JRE Men (n = 11)	0.961 (0.93–0.98)	2.16	21.0	5.99	58.2
JRE Women (n = 24)	0.733 (0.58–0.85)	4.24	37.7	11.75	104.5

°: degrees; %: percentage; ICC: Intra-class Correlation Coefficient; SEM: Standard Error Measurement SRD: Smallest Real Difference; CI: Confidence Interval; JRE: Joint Reposition Error. Bootstrapped 95% confidence intervals confirmed the stability of the ICC estimates.

## Data Availability

The original contributions presented in the study are included in the article; further inquiries can be directed to the corresponding author. The de-identified dataset containing paired test–retest JRE values that support the findings of this study is available from the corresponding author upon reasonable request. All relevant data are included within the manuscript.

## Abbreviations

The following abbreviations are used in this manuscript:

JPS	Joint Position Sense
JRE	Joint Reposition Error
ICC	Intraclass Correlation Coefficient
SEM	Standard Error of Measurement
SRD	Smallest Real Difference
CI	Confidence Interval
NPRS	Numerical Pain Rating Scale
IR	Interquartile Range
SPSS	Statistical Package for the Social Sciences
